# Mechanism of amyloid β−protein dimerization determined using single−molecule AFM force spectroscopy

**DOI:** 10.1038/srep02880

**Published:** 2013-10-07

**Authors:** Zhengjian Lv, Robin Roychaudhuri, Margaret M. Condron, David B. Teplow, Yuri L. Lyubchenko

**Affiliations:** 1 Department of Pharmaceutical Sciences, University of Nebraska Medical Center, 986025 Nebraska Medical Center, Omaha, NE 68198, United States; 2 Department of Neurology, David Geffen School of Medicine at UCLA, Los Angeles, CA 90095, United States; 3 Brain Research Institute and Molecular Biology Institute, David Geffen School of Medicine at UCLA, Los Angeles, CA 90095, United States

## Abstract

Aβ42 and Aβ40 are the two primary alloforms of human amyloid β−protein (Aβ). The two additional C−terminal residues of Aβ42 result in elevated neurotoxicity compared with Aβ40, but the molecular mechanism underlying this effect remains unclear. Here, we used single−molecule force microscopy to characterize interpeptide interactions for Aβ42 and Aβ40 and corresponding mutants. We discovered a dramatic difference in the interaction patterns of Aβ42 and Aβ40 monomers within dimers. Although the sequence difference between the two peptides is at the C−termini, the N−terminal segment plays a key role in the peptide interaction in the dimers. This is an unexpected finding as N−terminal was considered as disordered segment with no effect on the Aβ peptide aggregation. These novel properties of Aβ proteins suggests that the stabilization of N−terminal interactions is a switch in redirecting of amyloids form the neurotoxic aggregation pathway, opening a novel avenue for the disease preventions and treatments.

Aberrant folding (misfolding) and aggregation of amyloid proteins are pathological hallmarks of a large class of neurodegenerative diseases, including Alzheimer's disease (AD) (reviewed in^1^,^2^). In AD, amyloid precursor proteins are sequentially cleaved by β−secretase and γ−secretase, producing the amyloid β−protein (Aβ)^3^. Aβ peptides contain several alloforms with varying sequences of amino acids, dependent on the cleavage sites of γ−secretase. While Aβ40 is the most abundant species among these alloforms, Aβ42 is the most amyloidogenic species, and also appears to be the most toxic^4^,^5^,^6^. It was shown that the two extra C−terminal residues of Aβ42 dramatically change the peptide's oligomerization properties compared to Aβ40^7^,^8^. Aβ42 prefers to form a pentamer/hexamer structure (paranucleus) that can nucleate fibril formation^7^. By contrast, Aβ40 reaches rapid equilibrium with coexisting monomers, dimers, trimers, and tetramers^7^. According to computer simulations, Aβ42 has a turn−like fold at the C−terminus that involves Met35−Gly38 (referred to as a C−turn), suggesting that the more structured C−terminus of Aβ42 generates greater intramolecular contacts than Aβ40^9^. The hypothesis that the C−terminal residues play an important role in the Aβ aggregation process was recently tested by^10^, in which a set of Aβ peptides with defined alterations in the C−terminus were studied. Specifically, it was shown that VPV substitutions (Gly33Val−Val36Pro−Gly38Val) stabilized the β−hairpin structure by reducing the flexibility of the peptide backbone and by strengthening hydrophobic interactions between the putative β−strands. A similar VPV motif in an Aβ40 mutant changed the oligomerization pattern to one similar to Aβ42, resulting in increased toxicity of the oligomers compared with the toxicity of wild type (WT) Aβ40^10^. *pP* substitutions (Val36/*D*−Pro)−(Gly37/*L*−Pro) destabilized the putative C−turn structure and thereby made [*pP*]Aβ42 “Aβ40−like”^10^.

We have developed a single−molecule atomic force microscopy (SMFS) approach enabling us to probe and characterize the interactions of the peptides and proteins in transient misfolded conformations^11^,^12^,^13^,^14^,^15^. In this approach, the protein is end−immobilized on the AFM tip and substrate, allowing us to measure the interprotein interactions at the single−molecule level. The dynamics force spectroscopy (DFS) approach has been quite informative with respect to the lifetimes of transiently formed misfolded dimers (reviewed in^16^). These studies, applied to Aβ40^12^, Aβ42^14^, α−synuclein^13^ and Sup35^15^, revealed that misfolded dimers have an unexpectedly high stability. Their lifetimes exceeded the characteristic lifetimes of monomers by orders of magnitudes, suggesting that dimerization stabilized the misfolded protein state. Recent molecular dynamic (MD) simulations of Aβ(13−23) peptide directly support this view and show that the monomers adopt antiparallel β−sheet structures resulting in high dimer stability^17^. In addition to measuring the stability of dimers, SMFS allowed us to characterize the structure of the dimers and to identify the protein segments involved in dimer formation^11^,^12^,^13^,^14^. Using this approach, we showed that Cu^2+^ cations change the conformation of Aβ42 peptide, and dimer formation involves the N−terminal segments^14^. In the absence of the cations, Aβ42 peptides formed dimers via the C−terminal segments.

Here, we applied both SMFS approaches to characterize the effects of C−terminal substitutions on the structure of transiently formed dimers. The DFS approach revealed that VPV mutations in both Aβ40 and Aβ42 peptides resulted in stabilization. Additionally, the patterns of formation of misfolded Aβ dimers were studied by contour length analysis. These data provided an explanation of the stabilizing effect of the peptide C−terminus. We demonstrated that the interaction pattern of Aβ40 involving N−termini reduces dimers stability. These results are discussed in the context of the role of the N−terminus on the global structural reorganization of the misfolded Aβ dimers and their aggregation propensity.

## Results

### Single−molecule force spectroscopy

**Figure 1:**
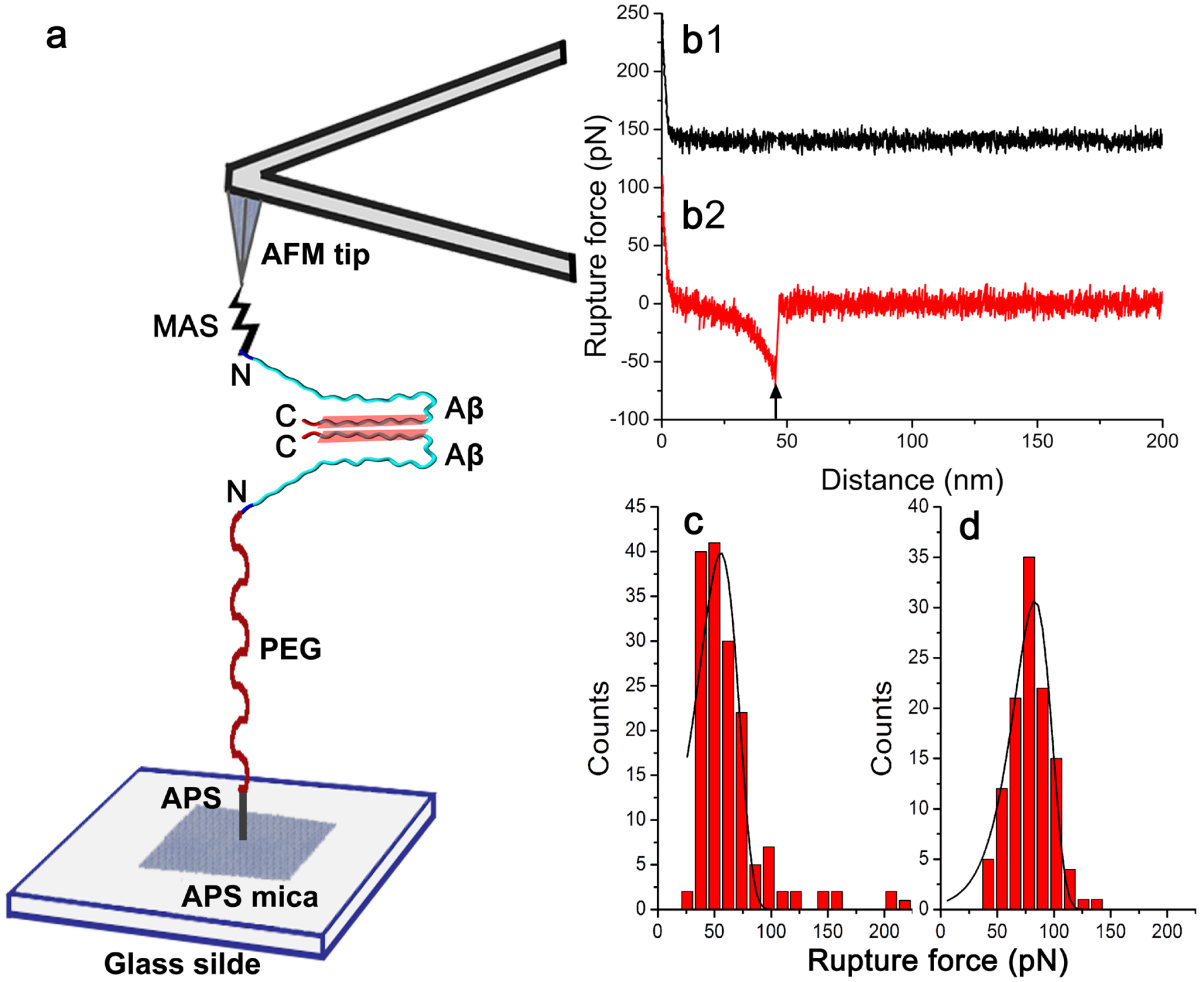
Schematic of the experimental setup. (a) One Aβ molecule immobilized on the mica substrate is picked up by another Aβ molecule functionalized on the AFM tip. Putative interaction segments within a dimer are highlighted in pink bands. A rupture event occurs when a misfolded dimer is torn apart. For clarity, only one linker molecule is drawn on AFM tip and substrate, and the sizes of objects in the scheme are not to scale. N and C denote N−terminus and C−terminus, respectively. (b) Typical force−distance curve that shows either no interactions (black) or specific interactions (red). In the case of specific interactions, breaking a misfolded Aβ dimer resulted in a rupture distance at ~ 48 nm and a rupture force at ~ 73 pN. A vertical black arrow indicates the rupture position. For clarity, only the retraction curves are shown. Y offset was performed with the red line but not with the black line. A typical histogram of rupture force distribution at a loading rate of 6215 pN/s for Aβ40 (c). The most probable rupture force for Aβ40 was 57.0 pN. A typical histogram of rupture force distribution at a loading rate of 6068 pN/s for [*VPV*]Aβ40 (d). The most probable rupture force for [*VPV*]Aβ40 was 83.6 pN. The solid lines represent the fit of probability density function.

The schematic of the SMFS experiments is shown in Fig. 1a. The cysteinyl−peptides were covalently immobilized on the tip and the surface via flexible tethers terminated with maleimide groups. Based on NMR and hydrogen−deuterium exchange experiments, Cys residues placed at the N−termini of the Aβ peptides should not significantly affect peptide structure, or aggregation^18^,^19^. Peptide aggregation was minimized by using a very low peptide concentration (20 nM) during the coupling reaction with the functionalized mica surface and the tips. Probing was performed by multiple approach−retraction cycles over various spots of the mica surface.

Figures 1−b1 and 1−b2 show typical force curves for non−specific and specific interactions, respectively. Specific rupture events were characterized by the appearance of the event indicated with an arrow in Fig. 1−b2. The rupture peak appears at the distance defined by the extension of the flexible linkers (polyethylene glycol, PEG and maleimide silatrane, MAS) and the segments of the peptides not involved in dimer formation (see Supplementary Methods for specifics). The force−distance curves, as shown in Fig. 1−b2, were analyzed with worm−like chain (WLC) fitting to extract parameters including the rupture force and the contour length. Rupture force distributions at specified loading rates were plotted and fitted with a probability density function, as demonstrated by the histogram in Fig. 1C obtained for Aβ40. The measurements for the [*VPV*]Aβ40 mutant performed at similar conditions (ionic strength, loading rate, etc.) are shown in Fig. 1d. Both histograms (1c and 1d) comprise narrow distributions of rupture forces, but the histogram 1d is shifted towards significantly higher rupture forces. The maxima of Gaussians were 83.6 pN and 57.0 pN for [*VPV*]Aβ40 and Aβ40, respectively, suggesting that the dimers formed by [*VPV*]Aβ40 are more stable than those formed by the WT Aβ40. The rupture forces increase with increasing apparent loading rates (Supplementary Fig. S1). To quantitatively characterize the properties of the dimers formed by Aβ40 and Aβ42 peptides, we employed the DFS approach, as described below.

### Dynamic force spectroscopy of misfolded Aβ dimers

In the DFS approach, multiple force measurements were performed at different pulling rates and the data were grouped into several clusters according to the selected loading rates. According to equation (3) (see Methods), the force data were linearly dependent on the logarithm of the loading rate. After extrapolating the data to the zero pulling rate, the intercept and the slope of the linear plots (DFS spectrum) produced characteristics of the complexes, including their stability (off−rate constant *k_off_*) and the energy barrier position (*x_β_*)^20^,^21^.

**Figure 2:**
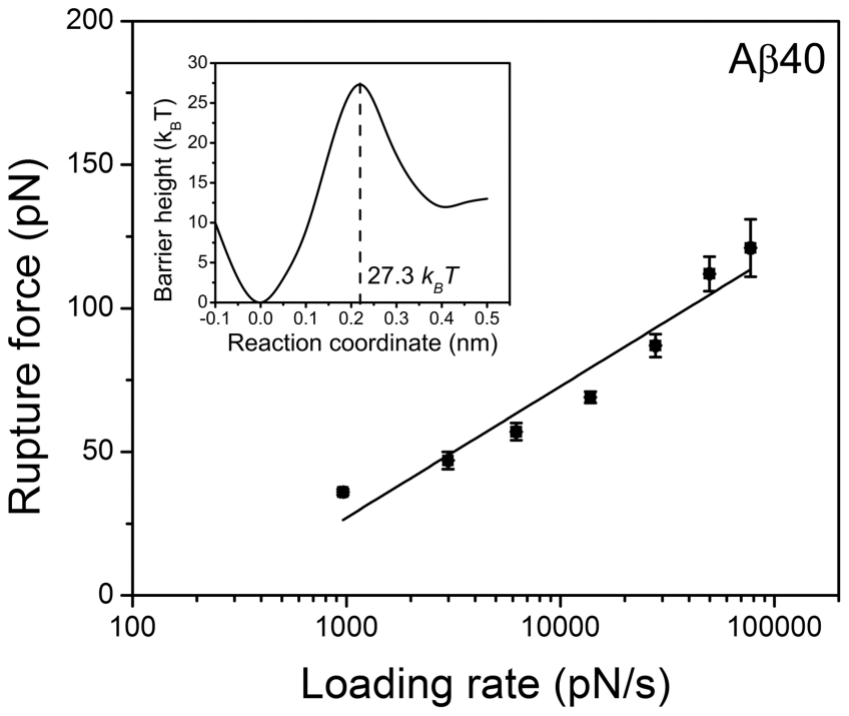
Representative dynamic force spectra of Aβ40. The solid red line represents the results fit with the Bell−Evans model. The obtained energy profile parameters are summarized in Table S1. The inset shows the reconstructed energy landscapes of misfolded Aβ dimers. Error bars represent ± SEM.

Figure 2 shows a DFS spectrum for Aβ40 peptide obtained from one experiment. The experiments were repeated three times, producing similar results. The *k_off_* and *x_β_* values determined from each separate experiment are shown in Supplementary Table S1. The mean values were calculated from this dataset (Supplementary Table S1) and equation (4) (see Methods) was used to extract the barrier height (Δ*G*), along with *x_β_* values, to generate the energy profile for the Aβ40 dissociation process. The profile is shown in the inset of Fig. 2.

Similar DFS spectra were obtained for [*VPV*]Aβ40 (Supplementary Fig. S2a), Aβ42 (Supplementary Fig. S2b), [*VPV*]Aβ42 (Supplementary Fig. S2c), and [*pP*]Aβ42 (Supplementary Fig. S2d) and the representative DFS plots for each mutant are shown in Supplementary Fig. S2. Each DFS was approximated by one linear plot, suggesting that the dissociation energy profiles have one barrier. The energy profiles were reconstructed from the mean values for *x_β_* and *k_off_* obtained from independent experiments for each peptide, shown in Supplementary Table S1. The corresponding energy profiles for each peptide are shown in the insets of Supplementary Fig. S2. The barrier heights were 27.3, 28.8, 27.7, 29.0 and 27.3 *k_B_T* for Aβ40, [*VPV*]Aβ40, Aβ42, [*VPV*]Aβ42 and [*pP*]Aβ42, respectively.

The stabilities of the dimers formed by the peptides were characterized by their lifetimes, (Supplementary Table S1). These data show that the initial stability of [*VPV*]Aβ42 dimers was more than three times higher than the wild type dimer (0.18 ± 0.01 s and 0.63 ± 0.12 s for Aβ42 and [*VPV*]Aβ42, respectively). A similar stabilization effect was observed for the Aβ40 peptide. The VPV mutation increased the lifetime from 0.11 s ± 0.03 for Aβ40 to 0.53 s ± 0.03 for [*VPV*]Aβ40. The pP mutation decreased the stability of [*pP*]Aβ42 dimers when compared to WT Aβ42 dimers (0.18 s ± 0.01 for Aβ42 vs. 0.11 s ± 0.01 for [*pP*]Aβ42). Overall, the stability of all five dimers decreased in the following order: [*VPV*]Aβ42 (0.63 s) > [*VPV*]Aβ40 (0.53 s) > Aβ42 (0.18 s) > [*pP*]Aβ42 (0.11 s) = Aβ40 (0.11 s). The data obtained for WT Aβ40 were similar to the recent data obtained at higher ionic strength (150 mM vs 10 mM 4)^12^, suggesting that changes in ionic strength in this broad range do not have pronounced effects on Aβ40 interactions.

### Characterization of misfolded states of Aβ dimers

We used the contour length measurements to identify segments of the peptides involved in dimer formation^12^,^13^,^14^. The measured contour length is comprised of the length of the flexible tethers used for peptide immobilization and the length of the segment of the peptide from the anchoring point to the rupture position. The latter, according to the schematic in Fig. 1, was obtained by subtracting the lengths of all linkers from the measured contour lengths.

**Figure 3:**
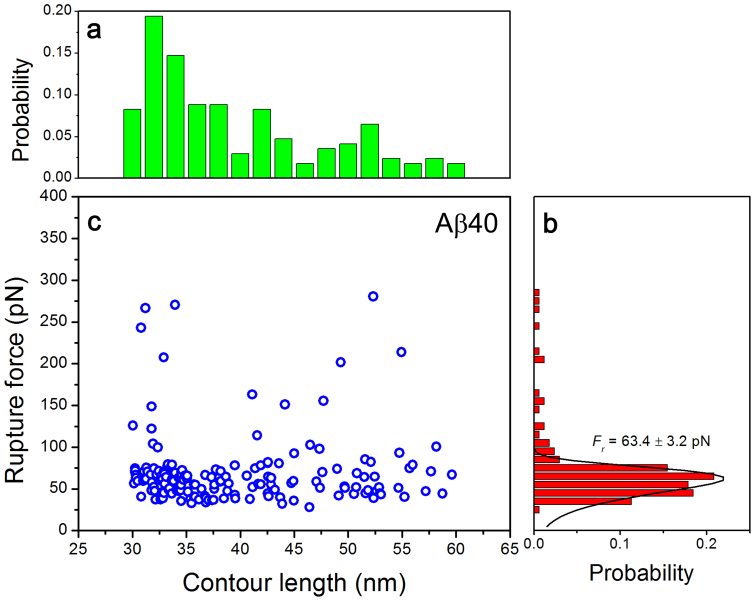
The distributions of contour length (a) and rupture force (b) at loading rates of 5000−7000 pN/s for Aβ40. The contour length distribution showed a major data cluster at ~ 33 nm. The most probable rupture force was 63.4 ± 3.2 pN. The scatter distribution of rupture forces with varying contour lengths is shown in (c). The total number of data was 170. Error is shown by ± SEM.

Figure 3a shows the contour length distribution for Aβ40 peptide obtained from 170 rupture events at loading rates of 5000−7000 pN/s. The distribution was broad but not smooth. The data primarily clustered around 33 nm, indicating that complexes dissociating from this pathway were the predominant species of Αβ40 dimers. The interactions via central and middle parts of the peptide exist, but their contribution is minor. The distribution of rupture forces from this dataset is shown in Fig. 3b. This histogram was rather narrow, with a most probable rupture force of 63.4 ± 3.2 pN, suggesting that the interaction strengths for dimers with different contour lengths are rather close, although minor events with forces beyond the main distribution also exist. The combined plot of rupture force versus contour length is shown in Fig. 3c. The data primarily aligned along a horizontal line at ~ 60 pN, with a limited number of rupture events with forces above this line appearing.

**Figure 4:**
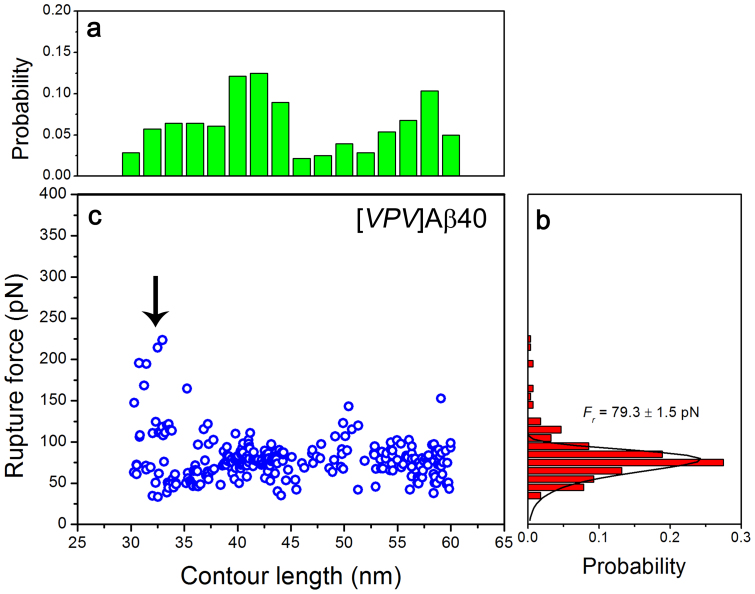
The distributions of contour lengths (a) and rupture forces (b) at loading rates of 5000−7000 pN/s for [*VPV*]Aβ40. The contour length distribution showed a major data cluster at ~ 42 and a comparable cluster at ~ 57 nm. The most probable rupture force was 79.3 ± 1.5 pN. The scatter distribution of rupture forces with varying contour lengths is shown in (c). The total number of data was 280. Error is shown by ± SEM.

A similar analysis performed for [*VPV*]Αβ40 is shown in Fig. 4. The contour length distribution of [*VPV*]Αβ40 (Fig. 4a) is broad, but entirely different form the one for the wild type Aβ40 (Fig. 3a). The distribution for [*VPV*]Αβ40 dimers has three clusters at ~ 33 nm, ~ 42 nm and ~ 57 nm that correspond to the interactions of distant C−terminal region of the peptide. Thus the major effect of VPV mutation is that the major data cluster at ~ 33 nm of Αβ40 dimers became the minor one of [*VPV*]Αβ40 dimers, whereas the other two data clusters of [*VPV*]Αβ40 dimers increased in size with approximately the same rate. The most probable rupture force of [*VPV*]Αβ40 dimers was 25% higher than that of Αβ40 dimers (79.3 vs 63.4 pN; Fig. 4b). Similar to Αβ40 dimers, the distribution of rupture forces of [*VPV*]Αβ40 dimers showed concentrated data points at low force levels (Fig. 4c), but had some doubled forces at contour lengths of ~ 32 nm (see arrow).

A similar contour length analysis was performed for Αβ42 and two mutants. The contour length distribution for Aβ42 is broad with the major contributions to the dimer stability from large contour length values ~ 56 nm (Supplementary Fig. S3a). VPV mutation shifts the distribution to the central region forming a major data cluster at ~ 42 nm with a substantial drop of distant rupture events (Supplementary Fig. S4a). The contour length distribution of the rupture of [*pP*]Αβ42 is entirely different form the above described distributions (cf. Supplementary Fig. S5a with Supplementary Figs. S3a and S4a). It has a maximum corresponding to small contour lengths values (~33 nm) and the entire distribution is very similar to the one for Aβ40 (Fig. 3a). Overall, the contour length distributions for Aβ40 and Aβ42 and their mutants are broad suggesting multiple populations of dimer structures. This finding is consistent with published MD simulation results^22^,^23^,^24^,^25^,^26^. Compared to the variability of contour lengths profiles for Aβ42 and the two mutants, the rupture force values are relatively similar. The most probable rupture force was 66.2 ± 0.8 pN for Αβ42 dimers (Supplementary Fig. S3b), 67.9 ± 1.0 pN for [*VPV*]Αβ42 dimers (Supplementary Fig. S4b), 68.0 ± 1.4 pN for [*pP*]Αβ42 dimers (Supplementary Fig. S5b), close to that of Αβ42.

## Discussion

The strong effect of the Aβ C−terminus on self−assembly is an intriguing property. The extra two amino acids of Aβ42 peptide are responsible for elevated neurotoxicity and a higher aggregation propensity than the Aβ40 peptide^27^. Here, we applied SMFS approach to investigate the interactions of inter− Aβ peptides. The SMFS analysis of Aβ peptides revealed that mutations of the very C−terminal regions caused significant differences in the propensity of the peptides to form transient dimeric species. The mutations: (1) change the stability of the dimeric species, and (2) alter the interaction pattern of the monomers within the dimers, leading to interactions a significant distance away from their C−terminal location.

The rupture force is a straightforward characteristic of interpeptide interactions, but this parameter is not very sensitive to C−terminal mutations of Aβ peptides. The DFS approach is a more sensitive technique than standard approaches and provides the ability to quantitatively characterize the effect of mutations. Our DFS results reveal that both [*VPV*]Aβ40 and [*VPV*]Aβ42 dimers are more stable than their WT counterparts (Fig. 2, Supplementary Fig. S2 and Table S1). These mutations increased the lifetimes of corresponding dimers by three fold: 0.53 s vs. 0.11 s for [*VPV*]Aβ40 vs. Aβ40 and 0.63 s vs. 0.18 s for [*VPV*]Aβ42 vs. Aβ42 dimers. By contrast, the [*pP*]Aβ42 dimers (0.11 s lifetime) are less stable than the WT Aβ42 dimers (0.18 s lifetime), which is also longer than the lifetime of Aβ40 dimers (0.11 s). Additionally, the energy profile of [*pP*]Aβ42 is analogous to that of Aβ40 dimers, as shown by the same lifetime (0.11 s for both [*pP*]Aβ42 and Aβ40).

According to previous simulation results^10^, the stabilization effect of VPV substitutions can be attributed to the constrained flexibility of the peptide backbone and the strengthened hydrophobic interactions at the C−terminus. The finding that Aβ42 dimers are more stable than Aβ40 dimers is consistent with the fact that Aβ42 is more prone to aggregation than Aβ40. The most probable rupture force of [*VPV*]Aβ40 is significantly higher than that of Aβ40 (Figs. 3 and 4). Combined with the longer lifetime of [*VPV*]Aβ40 dimers, these data indicate that VPV substitutions strengthen the interactions between Aβ40 peptides. However, this correlation is not true of Aβ42 peptides. The most probable rupture forces of WT Aβ42 and its mutants are similar (Supplementary Figs. S3−S5), but their lifetimes are different. These findings suggest that dimer characteristics other than rupture forces contribute to the stabilization effect of VPV mutations. The contour length measurements, as described below, were instrumental in unraveling these interaction patterns.

Probing interactions of Aβ peptides within a dimer by SMFS provides a pattern of peptide interactions within the dimers. According to the immobilization procedure, the peptide is covalently bound at its N−terminal cysteine. The experimentally measured contour length consists of two major components, the lengths of the flexible tethers used for peptide immobilization and the length of the peptide segment between the N−terminus and the segment of the peptide involved in interpeptide interactions. Therefore, the position of the peak on the force−distance curve is defined by the peptide length between the N−terminus and the interacting segment. If Aβ peptide adopts various conformations in these transient states, the positions of interacting segments, and hence the length of the non−interacting segments, will vary. The closer the interacting segment is to the N−terminus, the shorter the contour length will be when measured by AFM. The variable contour length measurements for all Aβ peptides in this study suggest that the monomers in the dimer adopt various conformations. The contour length of PEG 3400 was estimated to be 22.0 ± 0.9 nm^28^. Lengths of both aminopropyl silatrane (APS) and MAS were ~ 2.5 nm^13^. Therefore, the total length of all linkers was 24.5 ± 0.9 nm. In addition, the distance between N−termini of two peptides was estimated to be 1.0 nm.

**Figure 5:**
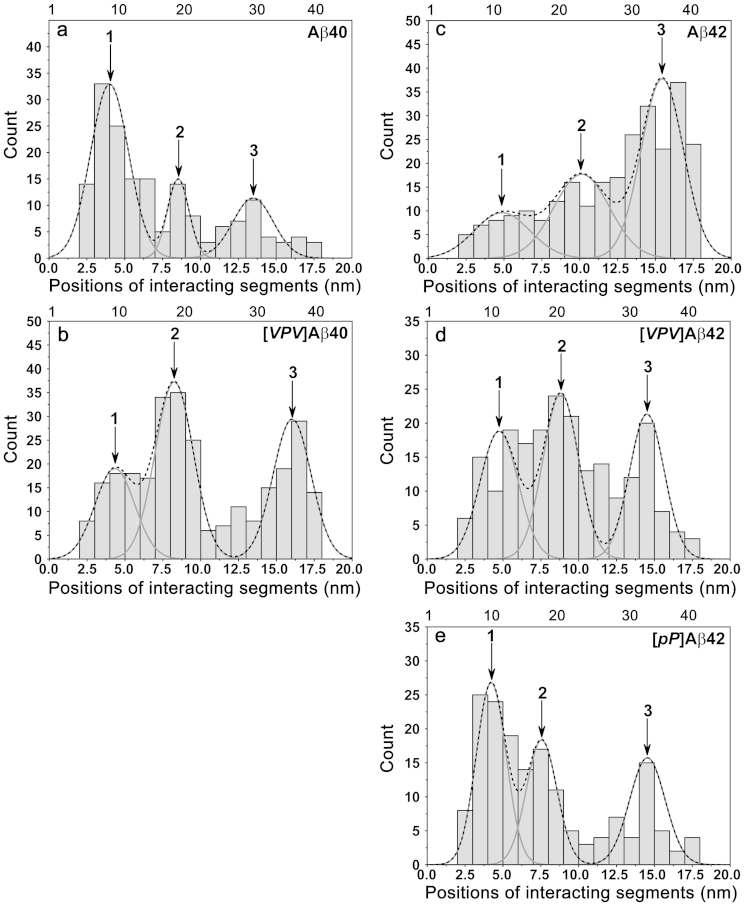
The contour length distributions for Aβ40, [*VPV*]Aβ40, Aβ42, [*VPV*]Aβ42, and [*pP*]Aβ42 dimers are shown in (a), (b), (c), (d) and (e), respectively. The interaction segments are aligned according to the contour length distribution. The dotted lines denote the distribution profiles and peaks are approximated according to the profiles. Notably, there are some uncertainties in length estimation. The arrows indicate interaction segments.

Figure 3 shows the contour length distribution for Aβ40 peptide. After subtracting the contour lengths of the tethers, the lengths of the stretchable peptide segments are obtained. The length distribution of the Aβ40 N−terminal segments is shown in Fig. 5a. This value was converted into the length in number of residues and is shown as the upper X axis on the graph. Our conversion values of 0.40 for Aβ42 and 0.41 for Aβ40 are close to the 0.38 nm value previously reported^29^,^30^. On this distribution, the predominant peak 1 corresponds to the segment of 1 ~ 10 residues, peak 2 corresponds to the longer segment of ~ 20 residues, and the third peak comprises almost 35 residues of the peptide from the N−terminus. This distribution suggests that Aβ40 can adopt at least three primary conformations within the dimers, which differ by the overall size of the peptide segments involved in the interpeptide interactions. The predominant conformation defined by peak 1 corresponds to the peptide conformation of a relatively short N−terminal segment. The rest of the peptide is involved with dimer formation. However, the interaction pattern changes dramatically if the VPV mutation is introduced into Aβ40 (Fig. 5b). Peak 1 exists, but it is not the predominant one, and peak 2 becomes larger. Additionally, there is a substantial increase in peak 3 that also moves to the very C−terminus of the peptide. Notably, this mutation leads to an increase in the rupture forces and the lifetime of the dimer. Combined with the change of the interaction pattern, these data suggest that the N−terminal sequence of the Aβ40 peptide dramatically alters the interaction pattern of the peptide in dimers and could modulate the toxicity of oligomers formed by the peptide.

A similar analysis was applied to the Aβ42 peptide and its mutants (Figs. 5c, 5d and 5e). First, we compared the contour length profiles of Aβ42 and Aβ40 peptides (Supplementary Figs. 5a and 5c). The extra two amino acids present in Aβ42 dramatically alter its interaction pattern compared to Aβ40. The primary interactions that occur via the N−termini for Aβ40 are replaced in Aβ42 by interactions within the C−terminus (Peak 3, Fig. 5c). This is consistent with a recent paper by Gu et al^31^, in which Aβ42 oligomers were found to be tightly packed at C−terminal region. Additional C−terminal modifications with triple substitutions (VPV mutations; Fig. 5d) result in further alterations in Aβ42 interaction. The most dramatic change observed in this mutant was the increase in interactions within the central part of the protein (observed by the growth of central peak 2) combined with decreased interactions of peptides via the C−termini (observed by the drop of peak 3). As mentioned above, the difference in dimer stability between Aβ42 and [*VPV*]Aβ42 as detected by the SMFS analysis was not due to the rupture forces because they were essentially similar. The contour length analysis explained the difference in the stabilities of Aβ42 and [*VPV*]Aβ42 dimers. The data in Figs. 5c and 5d suggest that a conformational change in the peptides occurs. As a result, the interpeptide interactions within the C−terminal region of Aβ42 (peak 3) are supplanted by interactions within the central segments of [*VPV*]Aβ42 (peak 2).

The Aβ42 double substitution of residues 36 and 37 with proline (*pP* mutations) results in very dramatic changes in the interpeptide interaction profile (*cf.* Figs. 5c and 5e). Overall, the interpeptide interaction is shifted to the N−terminus, as evidenced by the growth of peak 1. Surprisingly, the peptide interaction profile becomes similar to the profile for Aβ40 peptide (Fig. 5a). Notably, according to the DFS study (Supplementary Table S1), the stability of [*pP*]Aβ42 dimers as defined by their lifetime becomes as low as the lifetime for Aβ40 (0.11 s). Therefore, we hypothesize that the *pP* substitution causes [*pP*]Aβ42 monomer structures within the dimers to become similar to those for Aβ40 peptides.

It is important to compare our data with the aggregation studies of similar Aβ peptide samples. The studies performed by Roychaudhuri R et al^10^ showed that the aggregation properties of Aβ40 and [*pP*]Aβ42 are similar. Our analysis suggests that the similarity in aggregation is due to almost identical interaction profiles for both peptides. Similarly, mutating Aβ40 by VPV substitutions alters its aggregation propensity, causing it to be Aβ42−like. This effect is due to increased interactions between the central and C−terminal segments of [*VPV*]Aβ40 peptide dimers, compared to the predominate contribution of N−terminal interactions in Aβ40 dimers. This change in the interaction pattern also could explain the elevated neurotoxicity of [*VPV*]Aβ40 compared with the neurotoxicity of the WT peptide described in^10^. The model that the modulation of N−terminal segments of Aβ regulates toxicity is supported by the low toxicity of [*pP*]Aβ42 peptide aggregates^10^. V36*p*−G37P replacement makes [*pP*]Aβ42 interaction similar to Aβ40 interaction, with predominant interactions via N−terminal segments (cf. Figs. 5a and 5e). These data lead to a model of Aβ interaction suggesting that the N−terminal segments destabilize the dimer and thus decrease the aggregation propensity of the peptide and the cytotoxicity of the oligomers. *In silico* studies^10^ have suggested that the formation of the Val36−Gly37 turn is a key feature controlling Aβ42 aggregation and elevated neurotoxicity. Furthermore, VPV substitutions stabilize this structure. Our data show that stabilization of the turn is not a local structural change. Rather, interactions with other segments of the peptide are involved with both the stabilization of dimers and possibly higher order oligomers.

Our model on the effect of the N−terminal on the aggregation propensity of Aβ peptides is in line with the results of a recent paper^32^ in which the interaction of Aβ40 and Aβ42 monomers in solution was studied. They found that Aβ40 monomer forms a type of antiparallel β−hairpin involving the central hydrophobic cluster (residues 16−21) with the N−terminal residues 9−13, while Aβ42 monomer forms a major antiparallel β−hairpin involving the central hydrophobic cluster with the C−terminus. The contribution of N−terminal residues to the peptide interaction also reported in paper by Maji et al.^33^, in which effects of Tyr substitutions on the aggregation were studied. They report that Tyr substitution at Asp1 for both Aβ40 and Aβ42 leads to the slowest aggregation kinetics as opposed to Tyr substitutions at any other segments of the peptides.

A number of novel features of the interaction and self−assembly process of Aβ40 and Aβ42 peptides were determined using single−molecule force spectroscopy. The dynamic force spectroscopy analysis revealed stronger interpeptide interactions for Aβ42 than for Aβ40. The change in the peptide sequence at the C−terminus can increase or decrease dimer stability. VPV substitutions stabilize the Val36−Gly37 turn in Aβ42, whereas a *pP* dipeptide substitution destabilizes the dimer. A complementary single−molecule force spectroscopy approach, in which contour lengths were analyzed, revealed an unexpected long−range interaction effect in peptide dimerization. The N−terminal region of Aβ40 peptide cause its low interpeptide interaction, but the extra two residues of Aβ42 eliminate this effect. However, *pP* substitution in Aβ42 restores the effect of the N−terminus, therefore the stability of [*pP*]Aβ42 and Aβ40 become equal. The significance of this new finding on long−distance interactions within Aβ is two−fold. First, it demonstrates that Aβ peptide interaction patterns are complex and the seemingly neutral N−terminal region plays an important role in interpeptide interactions. Second, the involvement of the N−terminal region of Aβ in the interaction process suggests that this region may be a potential target for intervening into the Aβ misfolding process. These are novel avenues in the amyloid misfolding and aggregation area.

## Methods

All cysteinyl−Aβ peptides were synthesized using 9−fluorenylmethoxycarbonyl (FMOC) chemistry and purified by reverse phase high performance liquid chromatography (RP−HPLC). The identity and purity (usually > 97%) of the peptides were confirmed by amino acid analysis followed by mass spectrometry and RP−HPLC. Functionalization of AFM tips and mica substrates were prepared according to previously published procedures^11^,^12^,^14^. Briefly, 20 nM Aβ monomers were immobilized on mica functionalized with aminopropyl silatrane (APS) via PEG 3400 tethers and on AFM tips through maleimide silatrane (MAS). For DFS study, the tip retraction speeds ranged from 100 nm/s to 4000 nm/s. The dwell time was set at 0.3 s when retraction speed reached 1000 nm/s and above. Force curves with rupture event were subject to WLC analysis^12^,^14^, as shown below. 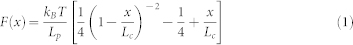
Rupture force distributions were fitted with a probability density function: 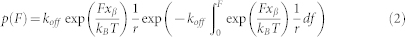
Dynamic force spectra were fitted with Bell−Evans model^21^: 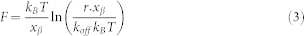
The energy profiles were reconstructed according to previous papers^20^,^34^, using the equation below. 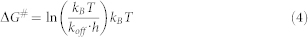
Positions of interacting segments of Aβ monomers within dimers were estimated by subtracting from the measured contour lengths the lengths of all linkers and the distance between N−termini of monomers (see Fig. 1a). The contour length of PEG was estimated with the following equation^28^: 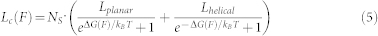
Where *L_c_(F)* is the contour length, *N_s_* is the average number of monomers, *L_planar_* is the length of monomers with planar conformation, *L_helical_* is the length of monomers with helical conformation, Δ*G(F)* is the free energy difference at zero appling force. The *N_s_* is 77 ± 10 for 3400 Da PEG. The *L_planar_* and *L_helical_* are 3.58 Å and 2.8 Å, respectively. The Δ*G(F)* is fixed at 3 *k_B_T*. The contour length of PEG was thus estimated to be 22.0 ± 0.9 nm. All error bars represent SEM. Detailed methods can be found in Supplementary Information.

## Author Contributions

Z.L., D.B.T. and Y.L.L. designed the experiments. R.R. and M.M.C. prepared initial samples. Z.L. performed the experiments and analyzed the data. Z.L., D.B.T. and Y.L.L. discussed the results and wrote the manuscript. All authors reviewed the manuscript.

## Supplementary Material

Supplementary Information Supplementary Information
