# Supplementary material for: Mechanism of amyloid β−protein dimerization determined using single−molecule AFM force spectroscopy
Source: Sci Rep. 2013 Oct 7;3:2880. doi: 10.1038/srep02880 (PMC3791449; doi:10.1038/srep02880)
Supplement: Supplementary Information [file srep02880-s1.pdf]

Mechanism of amyloid β−protein dimerization determined using single−molecule AFM force spectroscopy

Zhengjian Lv[1], Robin Roychaudhuri[2], Margaret M. Condron[2], David B. Teplow[2,3], Yuri L. Lyubchenko*[1]

[1] Department of Pharmaceutical Sciences, University of Nebraska Medical Center, 986025 Nebraska Medical Center, Omaha, NE 68198, United States

[2]Department of Neurology; and [3]Brain Research Institute and Molecular Biology Institute,

David Geffen School of Medicine at UCLA, Los Angeles, CA 90095

Supplementary Information

Supplementary Methods

Materials

The sequences of all five peptides were (substitutions were underlined):

Aβ40 (CDAEFRHDSGYEVHHQKLVFFAEDVGSNKGAIIGLMVGGVV);

Aβ42 (CDAEFRHDSGYEVHHQKLVFFAEDVGSNKGAIIGLMVGGVVIA);

[*VPV*] Aβ40 (CDAEFRHDSGYEVHHQKLVFFAEDVGSNKGAIIVLMPGVVV);

[*VPV*] Aβ42 (CDAEFRHDSGYEVHHQKLVFFAEDVGSNKGAIIVLMPGVVVIA);

[*pP*] Aβ42 (CDAEFRHDSGYEVHHQKLVFFAEDVGSNKGAIIGLMPPGVVIA). Each peptide included an additional residue (Cys) at its N−terminus to allow covalent attachment to the AFM tip or substrate. All peptides were synthesized using 9−fluorenylmethoxycarbonyl (FMOC) chemistry and purified by reverse phase high performance liquid chromatography (RP−HPLC). The identity and purity (usually > 97%) of the peptides were confirmed by amino acid analysis followed by mass spectrometry and RP−HPLC. Stock solutions of cysteinyl−Aβ peptides were prepared as previously described[1-3]. Briefly, for each Aβ peptide, the lyophilized form was dissolved in trifluoroacetic acid (2 mg/ml) by ultrasonication for 5 min to destroy preformed aggregates and then dried immediately using a vacuum centrifuge (Vacufuge, Eppendorf). The white powder of Aβ peptide was dissolved at 2 mg/ml in dimethyl sulfoxide (DMSO) as a stock solution and then diluted in DMSO before being used. The concentration of each Aβ peptide's stock solution was determined by spectrophotometry (Nanodrop® ND−1000). The molar extension coefficients were 1280 $cm^{-1}\cdot m^{-1}$ and 120 $cm^{-1}\cdot m^{-1}$ for tyrosine and cysteine, respectively. Aliquots of each peptide were stored at −20°C.

A 50 mM 1−(3−aminopropyl) silatrane (APS) stock solution was prepared by dissolving the APS powder in deionized (DI) water. The 1.67 mM stock solution of maleimide−polyethylene glycol−succinimidyl valerate (MAL−PEG−SVA; 3.4 kDa Laysan Bio Inc, Arab, AL) was prepared in DMSO (Sigma−Aldrich Inc.) and stored at −20°C. The 10 mM Tris (2−carboxyethyl) phosphine (TCEP) hydrochloride (Hampton Research Inc.) and the 2.94 mM solution of maleimide silatrane (MAS) were prepared in DI water and stored at −20°C. A 10 mM stock solution of β−mercaptoethanol was prepared in 10 mM sodium phosphate, pH 7.4 ("phosphate buffer") and kept at 4°C. Unless otherwise specified, other reagents used in the experiments were of analytical grade from Sigma−Aldrich. DI water (18.2 MΩ, 0.22 μm pore size filter, APS Water Services Corp., Van Nuys, CA) was used at all times.

Functionalization of AFM tips

The AFM tips were modified as described previously[1,4,5]. Briefly, silicon nitride (Si_3N_4) AFM tips (MSNL−10, Bruker Nano, Santa Barbara, CA) were immersed in 100% ethanol solution for 30 min, thoroughly rinsed with water, dried with argon, and then exposed to UV light (CL−1000 Ultraviolet Crosslinker, UVP, Upland, CA) for 30 min. The AFM tips were immersed in an aqueous solution of 167 μM MAS for 3 h and then rinsed with DI water. To monomerize Aβ molecules by breaking any intermolecular disulfide bonds, a 20 nM Aβ peptide solution in phosphate buffer was processed with 20 μM TCEP hydrochloride for 15 min before functionalization. The MAS−modified AFM tips were submerged into the above mentioned peptide solution for 1 h to covalently attach the peptides. After rinsing with pH 7.4 sodium phosphate buffer, the Aβ peptide−tethered AFM tips were treated with 10 mM β−mercaptoethanol solution for 10 min to quench the unreacted maleimide moieties. Finally, the

Aβ peptide–coated AFM tips were washed with phosphate buffer and stored in the same buffer solution. Typically, the storage time was less than 24 h at room temperature.

Modification of mica surfaces

Procedures for mica modification and peptide immobilization were similar to those described[1,4,5]. Briefly, mica sheets (Asheville–Schoonmaker Mica Co., Newport News, VA) were cut into 1.5 cm × 1.5 cm squares and glued to glass slides with epoxy. The freshly cleaved mica surfaces were treated with APS for 30 min followed by conjugation with 167 μM MAL–PEG–SVA in DMSO. After activation for 3 h, the mica squares were rinsed sequentially with DMSO and water to remove unbound MAL–PEG–SVA, and then dried with argon flow. The activated maleimide groups were then ligated with 20 nM cysteinyl–Aβ peptides for 1 h to make the Aβ –functionalized mica substrates. The remaining immobilization steps were the same as those described above for the AFM tips.

Dynamic force spectroscopy

The dynamic force spectroscopy force measurements were conducted with a Molecular Force Probe 3D AFM system (MFP–3D, Asylum Research, Santa Barbara, CA). AFM probes with nominal spring constants of 0.03 N/m were used throughout the experiments. The apparent spring constants were determined by the thermal noise analysis method with the Igor Pro 6.04 software (provided by the manufacturer). A low trigger force (100 pN) was exerted on the AFM probes. The approach velocity was constantly kept at 400 nm/s. To get a wide cover of loading rates, the retraction velocities of each DFS experiment were allowed to vary from 100 nm/s up to 4000 nm/s. When the retraction velocities reached 1000 nm/s or above, the dwell time was set

for 0.3 s. At each retraction velocity, force curves were obtained by probing over an area of 7×7 μm, creating force maps sized 60×40 points. The yield of rupture events varied between 2% and 4%. The nanomolar concentration of Aβ peptides was used at the peptide immobilization step resulting in a low surface density. The low surface density enabled us to avoid multiple interactions complicating the single molecule measurement. According to the paper of Merkel et al[6], 90% detection of specific interactions is achieved if 1 out of 7−10 attempts is a specific event. Generally, at least 100 force curves with typical rupture events were analyzed at each velocity to ensure reliable statistical analysis. Control experiments were conducted by eliminating the Aβ functionalization of either the tips or the mica substrates. Force curves with "rupture–like" events rarely occurred in control experiments. These force curves showed a "rupture" with a very short contour length (< 25 nm) and a small separation distance, an observation that could be attributed to nonspecific interactions between tips and linkers/substrates.

Estimation of the contour length of PEG 3400

The contour length of PEG was estimated with the following equation[7]:

$$L_c(F) = N_S \cdot \left(\frac{L_{planar}}{e^{\Delta G(F)/k_B T}+1} + \frac{L_{helical}}{e^{-\Delta G(F)/k_B T}+1}\right) \qquad (1)$$

Where $L_c(F)$ is the contour length, N_s is the average number of monomers, L_{planar} is the length of monomers with planar conformation, $L_{helical}$ is the length of monomers with helical conformation, $\Delta G(F)$ is the free energy difference at zero appling force. The N_s is 77 ± 10 for 3400 Da PEG. The L_{planar} and $L_{helical}$ are 3.58 Å and 2.8 Å, respectively. The $\Delta G(F)$ is fixed at 3 $k_B T$. The contour length of PEG was thus estimated to be 22.0 ± 0.9 nm.

Contour length analysis

As described previously[8], the data points in the loading rate range of 5000−7000 pN/s were used for the contour length analysis. The narrow range of loading rates was chosen to exclude the potential influence of different loading rates on the contour lengths. Throughout the contour length analysis, the arrangement of Aβ dimers was assumed to be symmetrical. This premise is reasonable based on work by Mitternacht *et al.*[9] in which near symmetrical Aβ42 dimers were confirmed by Monte Carlo simulation, and by the demonstration of the existence of symmetrical and in−register alignments of adjacent Aβ molecules within Aβ fibrils[10-12]. Based on the symmetrical model of Aβ dimers, the interaction positions where Aβ dimers dissociate were identified by the length calculation of all linkers. The contour length of PEG 3400 as described above is 22.0 ± 0.9 nm. Lengths of APS and MAS are 1.0 nm and 1.5 nm respectively[8]. Therefore, the total length of all linkers was 24.5 ± 0.9 nm. In addition, the distance between N−termini of the two peptides is ~1.0 nm. The contribution of the disordered segments of the peptides ranges from 0 nm to ~33 nm. The calculated combined contour length was divided equally between the Aβ monomers and was converted into the number of residues.

Dynamic force spectroscopy data analysis

Three rules were applied for identification of specific interactions[1,13]: (1) according to the thermal noise of the experimental setup, the rupture force larger than 20 pN were considered; (2) according to the control experiments, the contour length (the length at maximum physical extension of the interaction system determined after the worm−like chain (WLC) analysis) should be greater than 25 nm; and (3) the rupture events at distances of the tip−sample separation

(the projection of the distance between the AFM tip and the mica substrate on the vertical axis) smaller than 20 nm correspond to nonspecific interactions between the tip and these force curves were not analyzed.

The worm–like chain (WLC) model was used for fitting the force–distance curves[1,2,5]:

$$F(x) = \frac{k_B T}{L_p}\left[\frac{1}{4}(1-\frac{x}{L_c})^{-2} - \frac{1}{4} + \frac{x}{L_c}\right] \qquad (1)$$

where $F(x)$ is the force at the distance of x, k_B is the Boltzman constant, T is the absolute temperature, and L_p and L_c are the persistence length and the contour length, respectively. The persistence length of PEG was fixed at 0.35 nm[14]. The contour lengths were obtained with the Igor Pro 6.04 software package from the WLC fit of force–distance curves.

The apparent loading rates were calculated by using the following equation[5]:

$$\frac{1}{r} = \frac{1}{k_c v}\left(1 + \frac{k_c L_c}{4}\sqrt{\frac{F_p}{F^3}}\right) \qquad (2)$$

where $F_p = k_B T/L_p$, k_c is the spring constant (pN/nm), v is the tip retraction velocity, F is the rupture force, and r is the apparent loading rate (pN/s).

The most probable rupture force for each grouped data set was approximated with the probability density function[15]:

$$p(F) = k_{off}\exp(\frac{Fx_\beta}{k_B T})\frac{1}{r}\exp\left(-k_{off}\int_0^F \exp(\frac{Fx_\beta}{k_B T})\frac{1}{r}df\right) \qquad (3)$$

where $p(F)$ is the probability density of rupture force, k_{off} is the off–rate constant at zero external force, and x_β is the distance between the transition state and the bound state.

After obtaining the most probable rupture forces at a series of apparent loading rates, the dynamic force spectrum could be constructed and the corresponding data could be fitted with the Bell–Evans model[6]:

$$F = \frac{k_B T}{x_\beta} \ln(\frac{r \cdot x_\beta}{k_{off} k_B T}) \qquad (4)$$

Two important parameters, k_{off} and x_β, were thus extracted. The height of the energy barrier, $\Delta G^\#$, can be determined by the following equation[16,17]:

$$\Delta G^\# = \ln(\frac{k_B T}{k_{off} \cdot h}) k_B T \qquad (5)$$

where h is the Planck constant. All data errors were shown by ±SEM.

References

1 Lv, Z., Condron, M. M., Teplow, D. B. & Lyubchenko, Y. L. Nanoprobing of the Effect of Cu(2+) Cations on Misfolding, Interaction and Aggregation of Amyloid beta Peptide. *J Neuroimmune Pharmacol* **8**, 262-273, (2013).

2 Kim, B. H. *et al.* Single-molecule atomic force microscopy force spectroscopy study of Abeta-40 interactions. *Biochemistry* **50**, 5154-5162, (2011).

3 Walsh, D. M., Lomakin, A., Benedek, G. B., Condron, M. M. & Teplow, D. B. Amyloid beta-protein fibrillogenesis. Detection of a protofibrillar intermediate. *J Biol Chem* **272**, 22364-22372, (1997).

4 Yu, J., Malkova, S. & Lyubchenko, Y. L. alpha-Synuclein misfolding: single molecule AFM force spectroscopy study. *J Mol Biol* **384**, 992-1001, (2008).

5 Yu, J., Warnke, J. & Lyubchenko, Y. L. Nanoprobing of alpha-synuclein misfolding and aggregation with atomic force microscopy. *Nanomedicine* **7**, 146-152, (2011).

6 Merkel, R., Nassoy, P., Leung, A., Ritchie, K. & Evans, E. Energy landscapes of receptor-ligand bonds explored with dynamic force spectroscopy. *Nature* **397**, 50-53, (1999).

7 Oesterhelt, F., Rief, M. & Gaub, H. E. Single molecule force spectroscopy by AFM indicates helical structure of poly(ethylene-glycol) in water. *New Journal of Physics* **1**, 6, (1999).

8 Krasnoslobodtsev, A. V. *et al.* Effect of spermidine on misfolding and interactions of alpha-synuclein. *PLoS One* **7**, e38099, (2012).

9 Mitternacht, S., Staneva, I., Hard, T. & Irback, A. Monte Carlo study of the formation and conformational properties of dimers of Abeta42 variants. *J Mol Biol* **410**, 357-367, (2011).

10 Ahmed, M. *et al.* Structural conversion of neurotoxic amyloid-beta(1-42) oligomers to fibrils. *Nat Struct Mol Biol* **17**, 561-567, (2010).

11 Petkova, A. T. *et al.* A structural model for Alzheimer's beta -amyloid fibrils based on experimental constraints from solid state NMR. *Proc Natl Acad Sci U S A* **99**, 16742-16747, (2002).

12 Luhrs, T. *et al.* 3D structure of Alzheimer's amyloid-beta(1-42) fibrils. *Proc Natl Acad Sci U S A* **102**, 17342-17347, (2005).
13 Ray, C. & Akhremitchev, B. B. Conformational heterogeneity of surface-grafted amyloidogenic fragments of alpha-synuclein dimers detected by atomic force microscopy. *J Am Chem Soc* **127**, 14739-14744, (2005).
14 Gomez-Casado, A. *et al.* Probing multivalent interactions in a synthetic host-guest complex by dynamic force spectroscopy. *J Am Chem Soc* **133**, 10849-10857, (2011).
15 Friedsam, C., Wehle, A. K., Kühner, F. & Gaub, H. E. Dynamic single-molecule force spectroscopy: bond rupture analysis with variable spacer length. *Journal of Physics: Condensed Matter* **15**, S1709, (2003).
16 Krasnoslobodtsev, A. V., Shlyakhtenko, L. S. & Lyubchenko, Y. L. Probing Interactions within the synaptic DNA-SfiI complex by AFM force spectroscopy. *J Mol Biol* **365**, 1407-1416, (2007).
17 Tinoco, I., Jr. & Bustamante, C. The effect of force on thermodynamics and kinetics of single molecule reactions. *Biophys Chem* **101-102**, 513-533, (2002).

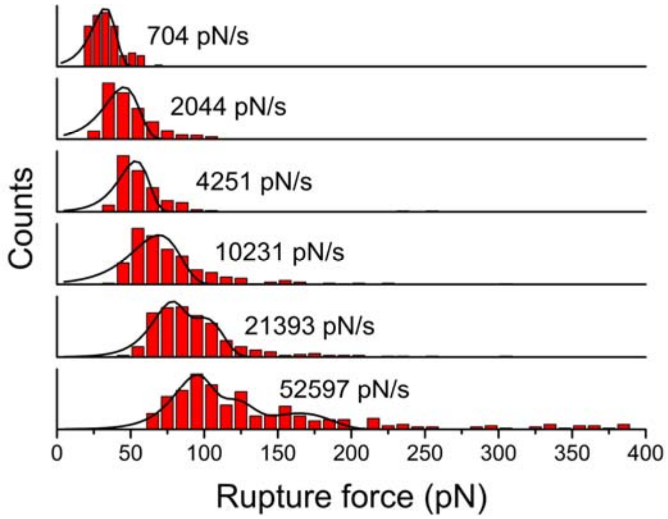

Supplementary Figure S1. A typical set of rupture force distributions for Aβ42 at different loading rates (the numbers shown in the inset). There was a clear trend that rupture forces increased with increasing loading rates. Probability density function was used to fit all distributions (solid lines) and to extract the most probable rupture forces.

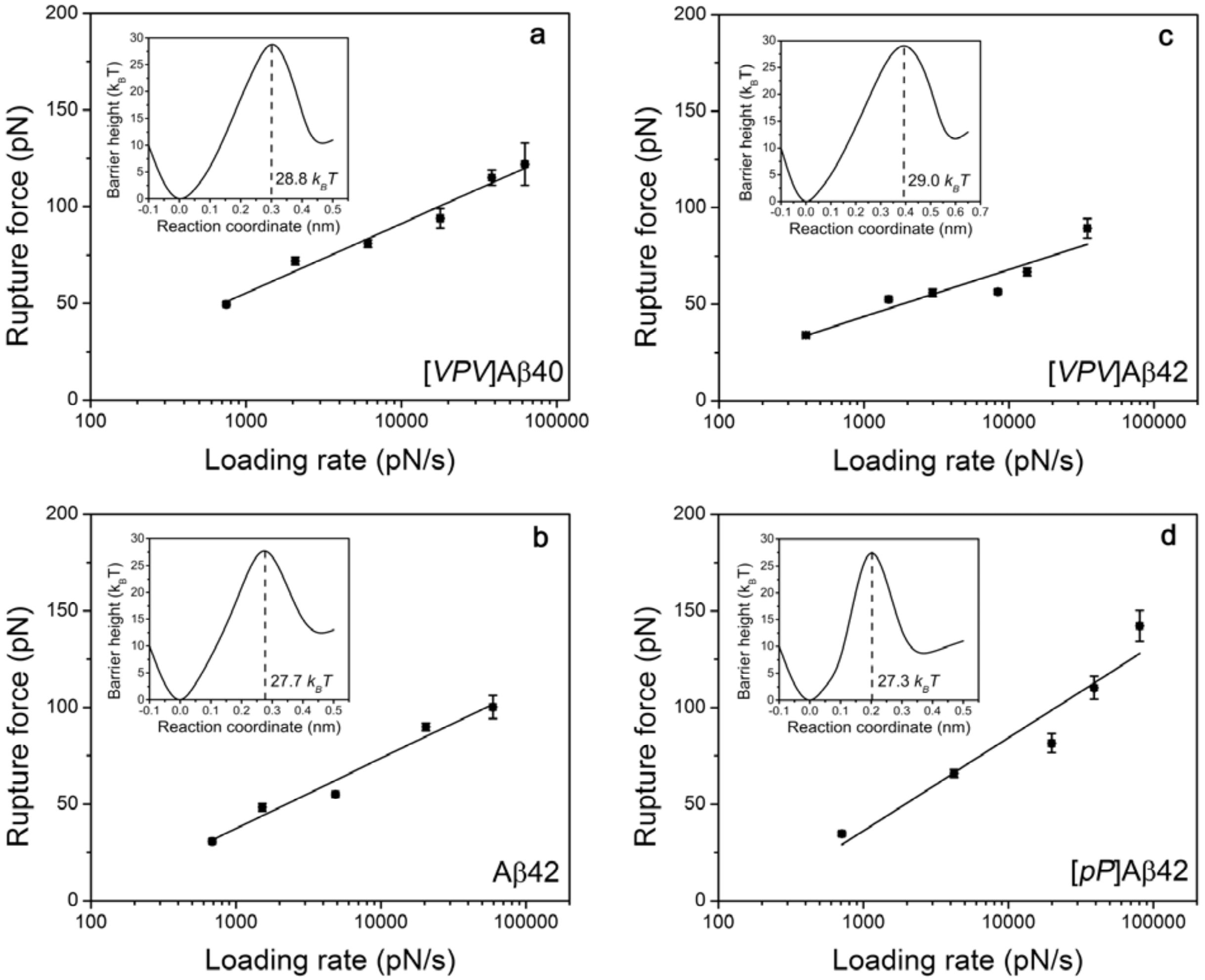

Supplementary Figure S2. Representative dynamic force spectra of [*VPV*]Aβ40 (a), Aβ42 (b), [*VPV*]Aβ42 (c), and [*pP*]Aβ42 (d). The solid red lines represent the results fit with the Bell–Evans model. The obtained energy profile parameters are summarized in Table S1. The insets show the reconstructed energy landscapes of misfolded Aβ dimers. Error bars represent ±SEM.

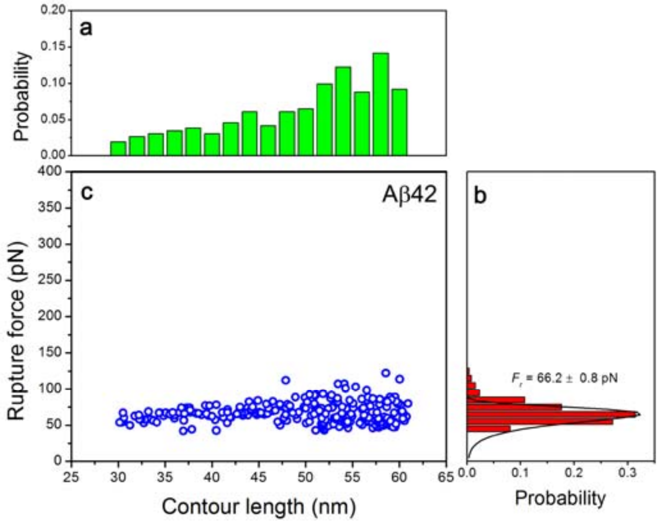

Supplementary Figure S3. The distributions of contour lengths (a) and rupture forces (b) at loading rates of 5000−7000 pN/s for Aβ42. The contour length distribution showed a major data cluster at ~56 nm. The most probable rupture force was 66.2 ± 0.8 pN. The scatter distribution of rupture forces with varying contour lengths is shown in (c). The total number of data was 261. Error is shown by ±SEM.

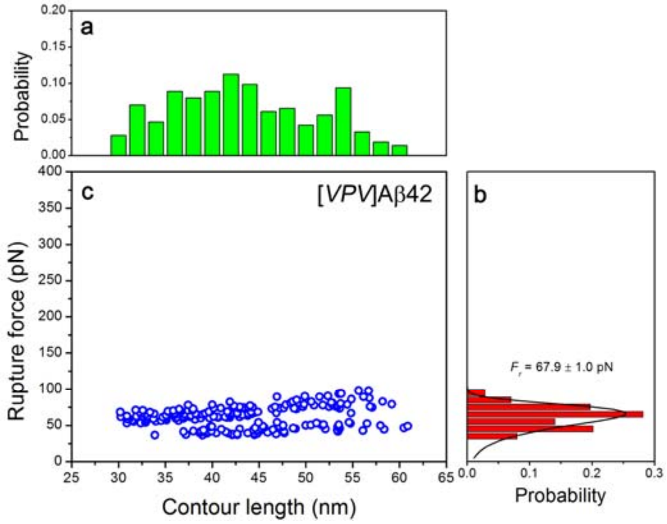

Supplementary Figure S4. The distributions of contour lengths (a) and rupture forces (b) at loading rates of 5000−7000 pN/s for [*VPV*]Aβ42. The contour length distribution showed a major data cluster at ~42 nm and a comparable cluster at ~54 nm. The most probable rupture

force was 67.9 ± 1.0 pN. The scatter distribution of rupture forces with varying contour lengths is shown in (c). The total number of data was 213. Error is shown by ±SEM.

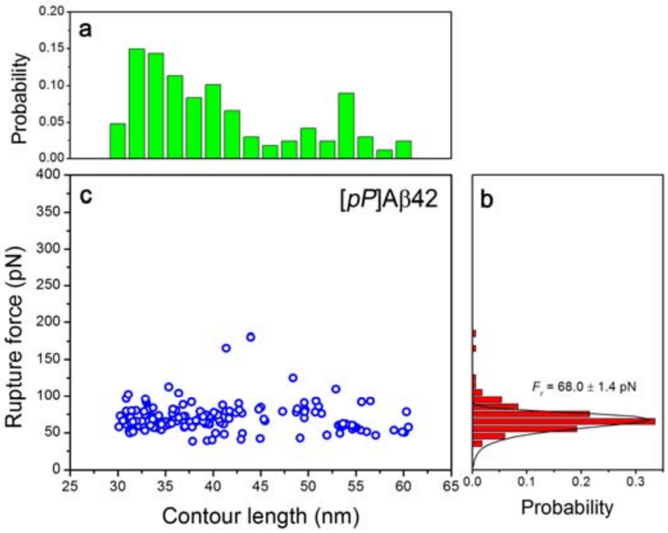

Supplementary Figure S5. The distributions of contour lengths (a) and rupture forces (b) at loading rates of 5000−7000 pN/s for [*pP*]Aβ42. The contour length distribution showed a major data cluster at ~33 nm. The most probable rupture force was 68.0 ± 1.4 pN. The scatter distribution of rupture forces with varying contour lengths is shown in (c). The total number of data was 167. Error is shown by ±SEM.

Supplementary Table S1. DFS data for all five dimers

	Exp	x_β (nm)	k_{off} (s^{-1})	Mean ± SE	τ (s)
	#1	0.203	9.4		
Aβ40	#2	0.257	4.7	x_β = 0.222 ± 0.018 nm	0.11 ± 0.03
	#3	0.206	12.9	k_{off} = 9.0 ± 2.4 s^{-1}	
	#1	0.361	2.2		
[*VPV*]Aβ40	#2	0.293	1.7	x_β = 0.305 ± 0.029 nm	0.53 ± 0.03
	#3	0.262	1.9	k_{off} = 1.9 ± 0.1 s^{-1}	
	#1	0.299	6.1		
Aβ42	#2	0.260	5.9	x_β = 0.274 ± 0.013 nm	0.18 ± 0.01
	#3	0.262	5.2	k_{off} = 5.7 ± 0.3 s^{-1}	
	#1	0.451	1.1		
[*VPV*]Aβ42	#2	0.337	2.1	x_β = 0.392 ± 0.034 nm	0.63 ± 0.12
	#3	0.387	1.5	k_{off} = 1.6 ± 0.3 s^{-1}	
	#1	0.160	9.6		
[*pP*]Aβ42	#2	0.196	8.5	x_β = 0.207 ± 0.031 nm	0.11 ± 0.01
	#3	0.265	7.9	k_{off} = 8.7 ± 0.5 s^{-1}	

x_β is the potential barrier location and k_{off} is the dissociation rate without applied force. The lifetime (τ) was calculated by $\tau = 1/k_{off}$. Exp denotes experiment. Errors are ±SEM.
